# Electrochemical and Mechanistic Study of Superoxide Elimination by Mesalazine through Proton-Coupled Electron Transfer

**DOI:** 10.3390/ph14020120

**Published:** 2021-02-04

**Authors:** Tatsushi Nakayama, Ryo Honda

**Affiliations:** 1 Department of Pharmacy, Gifu Pharmaceutical University, 1-25-4, Daigaku-nishi, Gifu 501-1196, Japan; 2 United Graduate School of Drug Discovery and Medical Information Sciences, Gifu University, 1-1 Yanagido, Gifu 501-1193, Japan; ryohonda.rh@gmail.com

**Keywords:** proton-coupled electron transfer, superoxide radical anion, mesalazine, cyclic voltammetry, electron spin resonance, ulcerative colitis

## Abstract

The elimination of superoxide radical anions (O_2_^•−^) by 5-amino-2-hydroxybenzoic acid (mesalazine, 5-ASA), 4-amino-2-hydroxybenzoic acid (4-ASA), and related compounds used for ulcerative colitis treatment was investigated using cyclic voltammetry and electron spin resonance (ESR) analyses aided by density functional theory (DFT) calculations. Quasi-reversible O_2_/O_2_^•−^ redox was found to be modified by the compounds, suggesting that an acid–base reaction in which a hydroperoxyl radical (HO_2_^•^) is formed from O_2_^•−^ occurs. However, the deprotonated 5-ASA anion can eliminate O_2_^•−^ through proton-coupled electron transfer (PCET), forming a radical product. This electron transfer (ET) was confirmed by ESR analysis. The 4-aminophenol moiety in 5-ASA plays an important role in the PCET, involving two proton transfers and one ET based on π-conjugation. The electrochemical and DFT results indicated that O_2_^•−^ elimination by 5-ASA proceeds efficiently through the PCET mechanism after deprotonation of the 1-carboxyl group. Thus, 5-ASA may act as an anti-inflammatory agent in the alkali intestine through PCET-based O_2_^•−^ elimination.

## 1. Introduction

5-amino-2-hydroxybenzoic acid (mesalazine, 5-ASA) is a medication used to treat inflammatory bowel conditions including ulcerative colitis and Crohn’s disease that can be taken orally or rectally [1,2]. As a non-steroidal anti-inflammatory drug (NSAID), its pharmacological mechanism is thought to be related to its anti-inflammatory action and the direct elimination of reactive oxygen species (ROS) [2,3,4,5]. 5-ASA was developed as a means to address the side effects associated with salazosulfapyridine (SASP), which is a prodrug of 5-ASA that was originally used to treat inflammatory conditions [1]. SASP is metabolized to the active 5-ASA moiety, which works by direct contact with the intestine, and sulfapyridine, which causes the side effects. Since 5-ASA is more easily degraded in the stomach than sulfapyridine, an enteric, pH-dependent coating that releases 5-ASA in the intestine without dissolving in the stomach is used.

It has been reported that both SASP and 5-ASA are poorly absorbed from the digestive tract [1]. This indicates that their therapeutic action is based on ROS elimination from the intestine and the surface of the gastrointestinal tract rather than cyclooxygenase inhibition after absorption, which is the main mode of action for typical NSAIDs. In addition, the reason that 5-ASA administered orally is not absorbed but instead degraded in the gastrointestinal tract is thought to be related to oxidation by ROS. Therefore, the mechanism of the reaction between 5-ASA and ROS, especially O_2_^•−^ as a precursor of several other ROS, must be considered when analyzing the ROS-elimination-based anti-inflammatory activity of 5-ASA and its degradation.

Several reaction mechanisms for O_2_^•−^ elimination by acidic substrates such as 5-ASA are known, including the superoxide-facilitated oxidation (SFO) [6,7,8], hydrogen–atom transfer (HAT) involving proton-coupled electron transfer (PCET) [9,10,11,12,13], and sequential proton-loss electron transfer (SPLET) [14]. In the SFO mechanism, the initial proton transfer (PT) from the substrate to O_2_^•−^ to give HO_2_^•^ is followed by rapid dismutation to give H_2_O_2_ and O_2_. Then, the substrate anion is oxidized by the O_2_ formed in the dismutation process [8]. Conversely, the other two mechanisms involve direct oxidation by O_2_^•−^ or HO_2_^•^.

Considering the possible mechanisms and structure–activity relationship of O_2_^•−^ elimination by 5-ASA, its 4-aminophenol (*p*-AP) moiety is thought to play a significant role in the PCET mechanism. In our previous studies, we reported that O_2_^•−^ is eliminated by polyphenols [11], diphenols (hydroquinone [10] and catechol [13]), and mono-phenols including *p*-AP [12] through the PCET mechanism. In these studies, the PCET mechanism for successful O_2_^•−^ elimination is based on quinone–hydroquinone π-conjugation and involves two PTs and one electron transfer (ET). Therefore, the *p*-AP moiety contained in 5-ASA is expected to act through PCET, although 5-ASA is also a salicylic acid and has a carboxyl group. It is presumed that the carboxyl group of 5-ASA makes its functionality different to that of *p*-AP, but its effect on O_2_^•−^ elimination through PCET is unclear.

Accordingly, in the present study we investigated the reaction between electrogenerated O_2_^•−^ and 5-ASA in *N*,*N*-dimethylformamide (DMF), focusing on the structural characteristics of 5-ASA. Furthermore, the mechanism of O_2_^•−^ elimination in relation to PCET has been electrochemically investigated with aid of density functional theory (DFT) calculations.

## 2. Results and Discussions

### 2.1. Cyclic Voltammetry Analysis of O_2_ in the Presence of 5-ASA and 4-ASA

**Figure 1:**
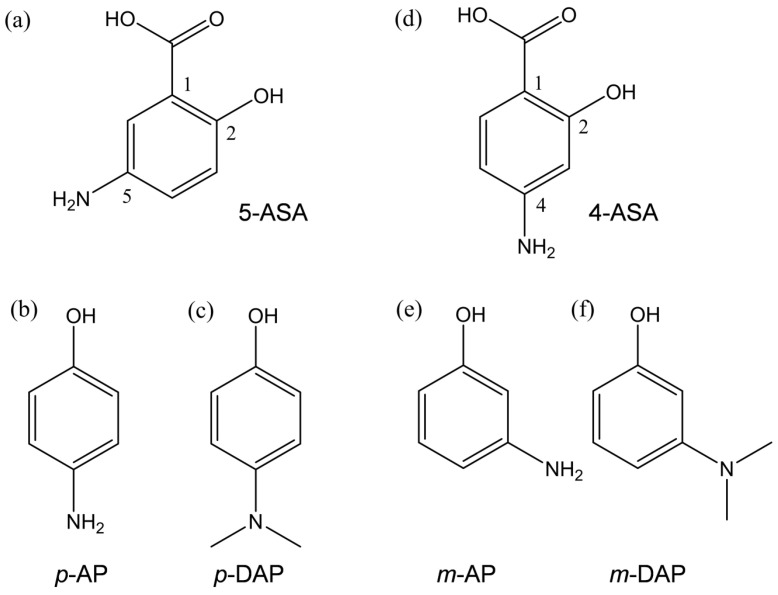
Structures of 5-ASA and related compounds considered in this study. (**a**) 5-amino-2-hydroxybenzoic acid (5-ASA), (**b**) 4-aminophenol (*p*-AP), (**c**) 4-dimethylaminophenol (*p*-DAP), (**d**) 4-amino-2-hydroxybenzoic acid (4-ASA), (**e**) 3-aminophenol (*m*-AP), and (**f**) 3-dimethylaminophenol (*m*-DAP).

**Figure 2:**
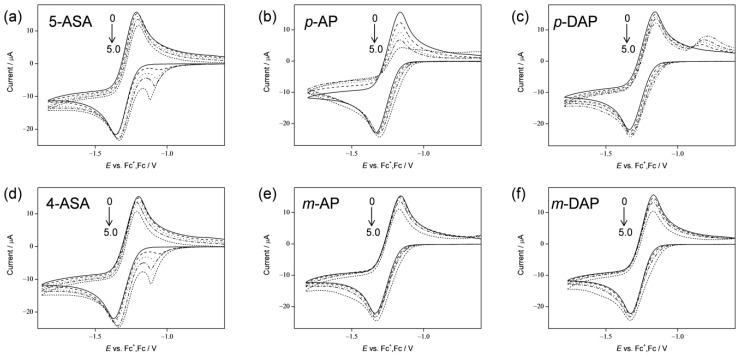
Cyclic voltammograms for 4.8 × 10^−3^ mol dm^−3^ O_2_ in the presence of (**a**) 5-ASA, (**b**) *p*-AP, (**c**) *p*-DAP, (**d**) 4-ASA, (**e**) *m*-AP, and (**f**) *m*-DAP in DMF containing 0.1 mol dm^−3^ tetrapropylammonium perchlorate (TPAP) recorded with a glassy carbon electrode at a scan rate of 0.1 V s^˗1^. Concentrations (×10^−3^ mol dm^−3^) are 0, 1.0, 2.0, 3.0, and 5.0. The concentration changes are indicated by arrows.

O_2_^•−^ elimination by 5-ASA, 4-ASA, and several related compounds (Figure 1) was investigated on the basis of electrochemical measurements in DMF. Figure 2 shows the cyclic voltammetry (CV) results for saturated O_2_ (4.8 × 10^−3^ mol dm^−3^) in the presence of the aminophenols (Ph(NH_2_)OH), 5-ASA, *p*-AP, 4-ASA, and *m*-AP; and the dimethylaminophenols, *p*-DAP and *m*-DAP. The voltammograms obtained in the presence of *p*-AP and *p*-DAP were reported in our previous paper, and are presented here for comparison [12]. In aprotic and unbuffered solvents such as DMF, O_2_ shows quasi-reversible redox (Equation (1)), corresponding to the generation of O_2_^•−^ in the initial cathodic scan and reoxidation to O_2_ in the returned anodic scan (solid lines, Figure 2a–f) [9,10,11,12,13,15]. The reversible CV behaviors investigated here are all modified to irreversible CV behaviors by the presence of all the compounds (a–f) in a concentration-dependent manner (0 to 5.0 × 10^−3^ mol dm^−3^), similar to that observed for typical phenolic compounds (Supplementary Information, Figure S1). This is supported by the fact that the CV traces in the absence of O_2_ (removed by bubbled N_2_) show no peaks over the potential range investigated (data not shown). Thus, the loss of reversibility in the O_2_/O_2_^•−^ couple is due to the initial PT from the compounds forming HO_2_^•^ (Equation (2)).

With the generation of HO_2_^•^, the bielectronic voltammograms observed are derived from the reduction of HO_2_^•^ (Equation (3)) as shown in Figure 2a,c–f. Conversely, in the presence of *p*-AP, the CV traces do not show bielectronic characteristics owing to the elimination of HO_2_^•^ by the subsequent ET (Equation (4)) from its phenolic anion (*p*-Ph(NH_2_)O^−^). In our previous study, the ET was experimentally observable by electron spin resonance (ESR) measurements owing to the radical product of the ET coupled with the second PT (Equation (5)), in which the *para*-amino group donates another proton [12]. However, the CV results obtained in the presence of 4-ASA and *m*-AP (Figure 2d,e) present bielectronic curves, which suggests that HO_2_^•^ (O_2_^•−^) is not eliminated through ET from the *m*-AP moiety.

These results confirm that the quinone–hydroquinone π-conjugated redox system [10,11] characteristic of the *p*-AP moiety plays an important role in PCET involving two PTs and one ET. Thus, for successful O_2_^•−^ elimination through PCET, two structural characteristics are required; (1) an amino proton for the second PT, and (2) *para*-substitution (i.e., the *p*-AP moiety) for π-conjugation.

O_2_ + e^−^ ↔ O_2_^•−^, *E*° = −1.284 V vs. Fc^+^/Fc (1)

O_2_^•−^ + Ph(NH_2_)OH → HO_2_^•^ + Ph(NH_2_)O^−^, the initial PT (2)

HO_2_^•^ + e^−^ → HO_2_^−^, *E*° = −0.4 to −0.2 V vs. Fc^+^/Fc (3)

HO_2_^•^ + *p*-Ph(NH_2_)O^−^ → HO_2_^−^ + *p*-Ph(NH_2_)O^•^, ET (4)

HO_2_^−^ + *p*-Ph(NH_2_)O^•^ → H_2_O_2_ + *p*-Ph(NH)O^•−^, the second PT (5)

**Figure 3:**
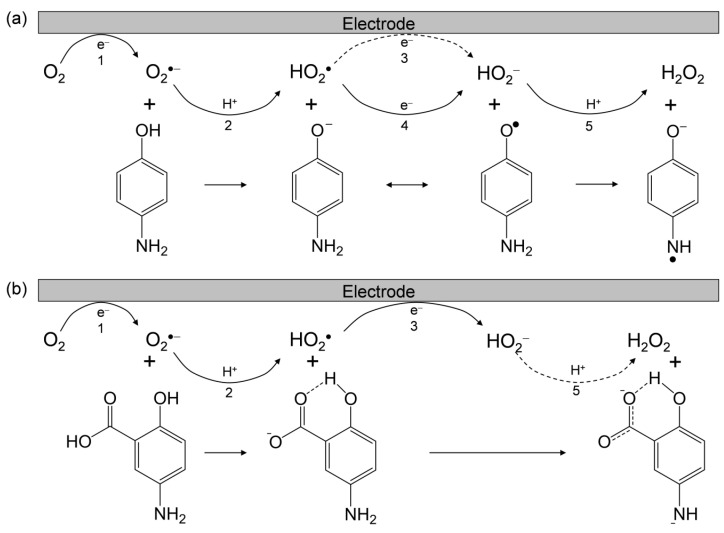
Plausible electrochemical mechanisms for O_2_/O_2_^•−^ in the presence of (**a**) *p*-AP and (**b**) 5-ASA in DMF. ^1^ One-electron reduction of O_2_/O_2_^•−^; ^2^ initial proton transfer (PT) from the acidic substrate to O_2_^•−^; ^3^ one-electron reduction of HO_2_^•^/HO_2_^−^; ^4^ electron transfer (ET) from the substrate anion to HO_2_^•^; ^5^ the second PT to HO_2_^−^.

Considering these results, we rationalized that O_2_^•−^ formation after the primary electrode process associated with PT from the phenolic hydroxyl group leads to the irreversible overall reduction of O_2_ to H_2_O_2_, which is driven by the exergonic reduction of the resulting HO_2_^•^/HO_2_^−^. Therefore, the CV traces for O_2_/O_2_^•−^ in the presence of phenolic compounds are divided into two typical curves: type A, an irreversible two-electron process observed in electro–chemical–electro (ECE) reactions (Equations (1)–(3)), and type B, an irreversible one-electron process (Equations (1), (2), (4) and (5)) leading to O_2_^•−^ elimination. Figure 3 shows the plausible electrochemical mechanisms for O_2_/O_2_^•−^ in the presence of *p*-AP and 5-ASA, summarizing Equations (1)–(5).

In this scenario, the CV results recorded in the presence *p*-AP demonstrate type B (elimination of O_2_^•−^). Conversely, the others involving both 5-ASA and 4-ASA demonstrate type A (O_2_^•−^ is not eliminated) showing the appearance of a cathodic current ascribed to HO_2_^•^. In Figure 2a,d, the reduction current for HO_2_^•^/HO_2_^−^ appears as a clear prepeak in the positive side of the O_2_/O_2_^•−^ peak due to the acidity of 5-ASA and 4-ASA derived from 1-carboxyl group. The voltammetric behavior for O_2_ in the presence of 5-ASA (and 4-ASA) is different from that of O_2_ in the presence of *p*-AP, despite the fact that 5-ASA contains a *p*-AP moiety in its structure. These CV results indicate that the 1-carboxyl group of 5-ASA suppresses ET via the PCET mechanism from the *p*-AP moiety and that single PT occurs from the carboxyl group instead. Furthermore, HO_2_^•^ formed by PT from the carboxyl group does not oxidize the *p*-AP moiety of the generated 5-ASA anion. Thus, this confirms that the ET is coupled with PT from a phenolic hydroxyl group or amino group, but not from the 1-carboxyl group. Therefore, the 1-carboxyl proton of 5-ASA inhibits PT from the phenolic hydroxyl group or amino group, resulting in the suppression of O_2_^•−^ elimination through the net PCET.

### 2.2. In Situ Electrolytic ESR Analyis of O_2_/O_2_^•−^ in the Presence of 5-ASA and the Related Compounds

**Figure 4:**
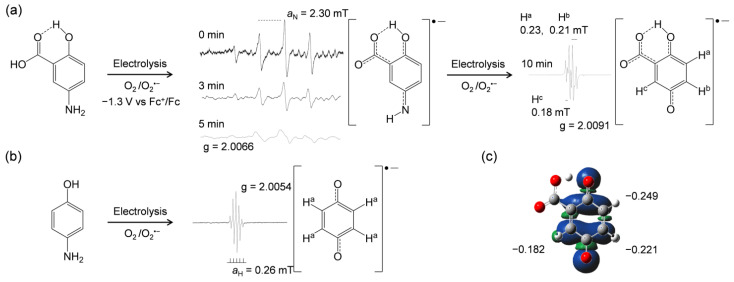
Electrolytic electron spin resonance (ESR) spectra for O_2_ (4.8 × 10^–3^ mol dm^–3^) in DMF solutions in the presence of 5-ASA (**a**), and *p*-AP (**b**) at 5.0 × 10^−3^ mol dm^−3^ as obtained by in situ controlled-potential electrolysis of solutions containing 0.1 mol dm^−3^ TPAP at an applied potential of −1.3 V vs. Fc^+^/Fc. The in situ ESR measurements were performed by 3 min scans 0, 3, 5, and 10 min after application of the potential began. Radical structures with the g-values and the appropriate hyperfine coupling constants (HFCCs) (mT) for nitrogen (*a*_N_) and hydrogen (*a*_H_) were obtained by simulation based on the measured spectra. The spin distribution for the 1,4-benzoquinone-2-carboxylic acid radical anion was calculated by density functional theory (DFT) at the B3LYP/PCM/6-311+G(d,p) level (**c**), and charge distributions for the 1-, 2-, and 5-position carbons were derived by natural bond orbital (NBO) analysis.

The ET from 5-ASA and the related compounds (Figure 1) to O_2_^•−^ and/or HO_2_^•^ has been inferred from in situ electrolytic ESR measurements. The ESR spectra of O_2_ solutions containing the compounds were measured using controlled-potential electrolysis at an applied potential of −1.3 V corresponding to O_2_ reduction (Equation (1)). ESR spectra were only obtained for the CV solutions containing 5-ASA or *p*-AP (Figure 4). As reported previously, the spectrum obtained for *p*-AP was assigned to 1,4-benzoquinone radicals based on the comparison of the experimental spectrum with the simulated hyperfine coupling constants (HFCCs) for hydrogen (*a*_H_) [12]. The ESR result shown in Figure 4b indicates that not only the ET but also an exchange of a nitrogen atom with an oxygen atom occurs in the *p*-AP moiety. However, the mechanism of this exchange is as yet unknown. Notably, ESR spectra were also obtained for 5-ASA (Figure 4a), despite no ET being indicated in the CV results (Figure 2a) at the timescale used. A spectrum with a large splitting at 2.30 mT, which may be derived from the amino nitrogen (*a*_N_), was initially observed (0 min; scanning initiated at the same time as voltage applied) upon ESR scanning for 3 min. However, this soon disappeared under the applied potential (see results obtained at 3 and 5 min) [16]. After 10 min of applied potential, another spectrum was presented, showing small splittings at 0.23, 0.21, and 0.18 mT.

Considering the ESR results for *p*-AP (Figure 4b), the *p*-AP moiety of 5-ASA is expected to react with O_2_^•−^ through PCET, because the initially observed ESR spectrum seems to be derived from a 1,4-quinone-imine-related radical. This result is significant in that it confirms the O_2_^•−^ elimination ability of 5-ASA, which is not observed for 4-ASA. Furthermore, the changes in the ESR spectrum suggest that the nitrogen atom of the 5-amino group exchanges with an oxygen atom by way of a 1,4-quinone imine radical. After this change, the ESR spectrum of the product can be assigned to the radical anion of 1,4-benzoquinone-2-carboxylic acid [17], as supported by the simulated results.

Next, the spin distribution on the radical product was calculated by DFT at the DFT-B3LYP/DMF/6-311+G(d,p) level, and the results are shown in Figure 4c. Furthermore, the charge numbers corresponding to the spin electron were obtained by the natural bond orbital (NBO) analysis (Supplementary Information, Table S1). The calculated charges on the 1-, 2-, and 5-position carbons of the quinone radical moiety denoted in Figure 4c are in good correlation with the HFCCs observed in the experimental ESR results for the product radical. Based on these results, the HFCCs were assigned as H^a^: 0.23, H^b^: 0.21, and H^c^: 0.18 mT, accurately identifying the product radical structure.

As shown above, the 1-carboxyl group suppresses PCET at the CV timescale by donating the carboxyl proton to O_2_^•−^, forming the deprotonated 5-ASA anion. However, this deprotonation enables subsequent PCET from the *p*-AP moiety to another O_2_^•−^. Therefore, it is reasonable to assume that the reaction product is a 1-carboxyl benzoquinone radical, formed through sequential 1:2 reactions, the initial PT of the 1-carboxyl proton to O_2_^•−^, and the subsequent PCET between another O_2_^•−^ molecule and the *p*-AP moiety followed by exchange of the nitrogen atom with an oxygen atom. These experimental results demonstrate that 5-ASA can eliminate O_2_^•−^ through PCET in basic DMF solutions.

### 2.3. Stable-Structure Optimization for 5-ASA and Its Anion by DFT Calculation

**Figure 5:**
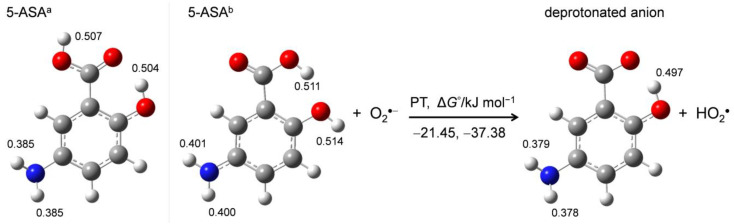
Different conformers of 5-ASA (5-ASA^a^, 5-ASA^b^) and their deprotonated anion showing the initial PT calculated with DFT-B3LYP/PCM/6-311+G(d,p) in DMF. Calculated Δ*G*°s (kJ mol^−1^) for the PT and the charges distributed on the protons obtained by the NBO analysis are denoted.

DFT calculations were performed to aid the mechanistic analysis of O_2_^•−^ elimination by 5-ASA. First, in order to obtain the structures of the stable conformer of 5-ASA and its corresponding anion, energy scanning for the dihedral angle around the 1-carboxyl group was performed. Figure 5 shows the optimized structures of two 5-ASA conformers (5-ASA^a^, 5-ASA^b^) and the deprotonated anion upon initial PT. Calculated standard Gibbs free energy changes (Δ*G*°/kJ mol^−1^, 298.15 K) for the PT and distributed charges on the acidic protons obtained by NBO analysis (Supplementary Information, Table S2) are denoted.

5-ASA has two planar conformers that differ in the orientation of the 1-carboxyl group, forming an intramolecular hydrogen bond (HB) between adjacent 2-hydroxyl groups. Comparison of the charge distribution on the acidic protons shows that the 1-carboxyl and 2-hydroxyl protons are more positively charged than the 5-amino protons, suggesting that the 1-carboxyl or 2-hydroxyl proton is transferred in the initial acid–base reaction to an alkali O_2_^•−^. The Δ*G*° values show that 5-ASA^a^ is 15.93 kJ mol^−1^ more stable than 5-ASA^b^ in DMF, though the PT from 5-ASA^a^ (−21.45) and 5-ASA^b^ (−37.38) are exergonic reactions. For either conformer, the proton that is not involved in the intramolecular HB that is transferred to O_2_^•−^, forming a conformer of the corresponding anion that is stabilized by the intramolecular HB [18], as shown in Figure 5. These calculation results demonstrate that the initial PT occurs from the 1-carboxyl or 2 hydroxyl group forming the stable anion, which involves the *p*-AP moiety having an intramolecular HB.

### 2.4. Change in HOMO–LUMO Energies upon PCET Between O_2_^•−^ and 5-ASA

**Figure 6:**
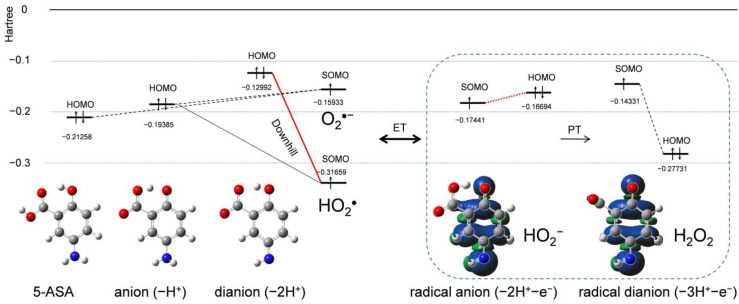
Changes in HOMO–LUMO energies (Hartree) upon proton-coupled electron transfer (PCET) between O_2_^•−^ and 5-ASA in DMF, calculated with the DFT-B3LYP/PCM/6-311+G(d,p) method.

For a detailed analysis of the PCET involved in O_2_^•−^ elimination by 5-ASA, HOMO–LUMO changes upon PCET between O_2_^•−^ and 5-ASA were calculated by the DFT method (Figure 6). After the initial PT, some reactant species, i.e., 5-ASA, the deprotonated (−H^+^) 5-ASA anion, the dianion, O_2_^•−^, and HO_2_^•^ coexist in the solution. The SOMO energy (Hartree) for HO_2_^•^ (−0.31659) is much lower than HOMO energies of 5-ASA, the anion, and the dianion. Thus, the electron acceptor will be HO_2_^•^, not O_2_^•−^. Considering that CV revealed that HO_2_^•^ is not eliminated by the 5-ASA anion (Figure 2a), the electron donor will be the dianion, for which the downhill energy relationship is indicated by the bold red line in Figure 6. Thus, this change in HOMO–LUMO energies upon PT between the 5-ASA anion and O_2_^•−^ forming the dianion and HO_2_^•^ is reasonable for the subsequent ET. Furthermore, the fact that the ET from the 5-ASA anion to HO_2_^•^ was not observed in the CV results and the downhill HOMO–LUMO energies implies that the PT and ET occur concertedly, as the amino group with adjacent ring can donate both a proton and an electron.

The HOMO–LUMO relationship between the products after ET (i.e., the 5-ASA radical anion and HO_2_^−^) is reversed, which is rational for orbital energies in the reverse ET (red dotted line in Figure 6). However, the HOMO (−0.27731) of the PT-forming hydroperoxide (H_2_O_2_) is lower than the HOMO (−0.16694) of HO_2_^−^, making the reverse ET impossible. Thus, the subsequent PT is dominant in determining the ET direction.

Analysis of the HOMO–LUMO relationship for O_2_^•−^ elimination by 5-ASA indicates that the concerted PCET involving two PTs and one ET occurs between the 5-ASA anion and O_2_^•−^ after initial deprotonation of the 1-carboxyl group. As reported previously, a concerted PCET occurs after the formation of the pre-reactive hydrogen-bonded complex from the free reactants, and the proton and electron are transferred in one kinetic step via a transition state forming the product complex [11,13]. Therefore, it is expected that the PCET between the 5-ASA anion and O_2_^•−^ occurs in a similar concerted mechanism through the HB formed between the amino group of the *p*-AP moiety and O_2_^•−^.

### 2.5. Free-Energy Analysis of PCET between Electrogenerated O_2_^•−^ and the Deprotonated 5-ASA Anion

**Figure 7:**
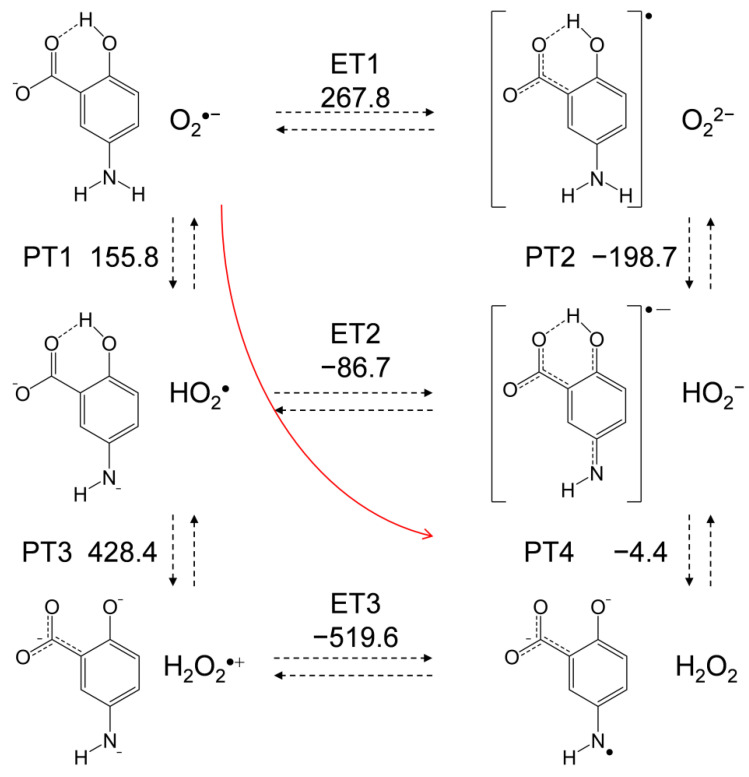
Six diabatic electronic states and the Δ*G*° values for the PCET between O_2_^•−^ and the deprotonated 5-ASA anion involving two PTs and one ET. Δ*G*° (kJ mol^−1^, 298.15 K) for the PT (PT1–PT4) and ET (ET1–ET3) were calculated using the DFT-(U)B3LYP/PCM/6-311+G(d,p) method.

In Figure 7, an equilibrium scheme and Δ*G*° values for the six diabatic electronic states in the PCET between the deprotonated (−H^+^) 5-ASA anion and O_2_^•−^ as calculated using DFT are shown. Since ET between HO_2_^•^ and the 5-ASA anion was not observed by CV, ET1 corresponding to oxidation of the 5-ASA anion by O_2_^•−^, which has less oxidative power than HO_2_^•^, is not feasible. Thus, PT1 must occur first, although both ET1 and PT1 are uphill endergonic reactions (Δ*G*° = 267.8 and 155.8 kJ mol^˗1^, respectively). In the following pathways, though PT3 is also endergonic, an exergonic ET2 (−86.7) followed by PT4 (−4.4) is a feasible pathway. Then, PT4 is expected to follow ET2 because of its exergonic Δ*G*° (−4.4) (although the ESR spectrum observed for the reaction products could not unambiguously identify the terminal of the PCET pathway). In our previous study [12], we determined that the second PT from the aminophenol moiety is necessary for the PCET, as is also demonstrated by the CV results (Figure 2c), which revealed that *p*-DAP, which has no amino proton, does not bring about successful HO_2_^•^ elimination. Additionally, analysis of the HOMO–LUMO relationship between 5-ASA and O_2_^•−^, as shown in Figure 6, indicates that the subsequent PT is the dominant factor that determines the ET direction. These findings imply that PT1 coupled with ET2–PT4 proceeds in a concerted manner without generating high energy intermediates, corresponding to the red line shown in Figure 7.

For a comparative study, the Δ*G*° values of the PCET pathways for *p*-AP, the 4-ASA anion, and *m*-AP were calculated (Table 1). The total values of Δ*G*° for the net PCET were obtained from the sum of the values for two PTs and one ET. From a thermodynamic viewpoint, the total values embody the energetic driving force of the net PCET for the 5-ASA anion and *p*-AP. Conversely, the total value for the 4-ASA anion (214.4) cannot embody the energetic driving force because the Δ*G*° for the unfeasible single ET has been summed in it. The important factor for the net PCET pathway comprised of PT1–ET2–PT4 to proceed as a sequential reaction is the Δ*G*° values of the individual reactions. The acid−base interaction and the redox potentials of the components, rather than the total Δ*G*°, are exergonic. In the pathway, the Δ*G*° of PT4 (118.3) for the 4-ASA anion constitutes an uphill energy barrier to the net PCET reaction, and the same is true for that of ET2 (15.2) for *m*-AP. Thus, these Δ*G*° values confirm that PCET is feasible for the *p*-AP moiety but not for the *m*-AP moiety, supporting the other experimental results.

For the 5-ASA anion and *p*-AP, PT1 is an uphill energy process, but the subsequent ET2 and PT4, are downhill. Therefore, a sequential pathway (PT1–ET2–PT4) is possible while the latter downhill reactions promote the net reaction equilibria. Alternatively, a concerted two-proton-coupled electron transfer (2PCET) may be a more feasible pathway than sequential PCET owing to its kinetic advantage (Figure 7, red line). For the concerted pathway, PT1 will proceed with more efficient kinetics than single PT1 for sequential PCET involving one concerted kinetic step comprising ET2 and PT4. Furthermore, it should be noted that the subsequent reaction after the PCET equilibria is the irreversible decomposition of the product quinone radical via an intermediate amino radical, so the net PCET will be promoted.

## 3. Materials and Methods

### 3.1. Chemicals

5-ASA, 4-ASA, *p*-AP, *m*-AP, and *m*-DAP were obtained from Sigma–Aldrich Inc (Tokyo, Japan). *p*-DAP was obtained by methylating *p*-AP. The methylation was conducted using dimethyl sulfate as an electrophilic methyl source, and the obtained filtrate was concentrated and separated using a thin-layer chromatography plate (benzene, silica gel 60 F_254_, 2 mm) [19]. All the chemicals were recrystallized from benzene and dried sufficiently under reduced pressure before use. The solvent for electrochemical and ESR measurements was spectrograde-purity DMF available from Nacalai Tesque, Inc. (Kyoto, Japan), which was used as received. TPAP for a supporting electrolyte in DMF was prepared as described previously [20]. Ferrocene (Fc), used as a potential reference compound, was obtained from Nacalai Tesque Inc. (Kyoto, Japan) and purified by repeated sublimation under reduced pressure immediately prior to use.

### 3.2. Electrochemical and In Situ Electrolytic ESR Measurements

CV was recorded via a three-electrode system comprising a glassy carbon (GC) working electrode, a coiled platinum counter electrode, and an Ag/AgNO_3_ reference electrode (containing a CH_3_CN solution of 0.1 mol dm^−3^ TBAP and 0.01 mol dm^−3^ AgNO_3_; BAS RE-5) at 25 °C using a BAS 100B electrochemical workstation coupled to BAS electrochemical software. The reference electrode was calibrated with reference to the ferrocenium ion/ferrocene couple (Fc Fc^+^/Fc), and all potentials reported here are referenced to the potential of this couple. In situ electrolytic ESR spectra were measured using a JEOL JES-FA200 X-band spectrometer. The controlled potential electrolysis was performed at room temperature using an electrochemical ESR cell with a 0.5 mm diameter straight Pt wire sealed in a glass capillary as a working electrode (Supplementary Information, Figure S2).

Samples were prepared in a glove box completely filled with N_2_ gas to prevent contamination by moisture. A DMF solution containing 0.1 mol dm^−3^ TPAP as a supporting electrolyte was saturated with O_2_ by air-bubbling the gas for ca. 2−3 min, and the gas was passed over the solutions during the electrochemical and ESR measurements to maintain the concentration of O_2_ at a constant level. The equilibrium concentration of O_2_ was calculated as 4.8 × 10^−3^ mol dm^−3^.

### 3.3. Calculations

All solution phase calculations were performed at the DFT level with the Becke three-parameter Lee–Yang–Parr (B3LYP) hybrid functional as implemented in the Gaussian 16 Program package [21]. The geometry optimization, subsequent vibrational frequency calculations, and population analysis of each compound were carried out by employing the standard split-valence triple ζ basis sets augmented by polarization d,p and diffusion orbitals 6-311+G(d,p). The solvent contribution of DMF to the standard Gibbs free energies was computed employing the polarized continuum model (PCM) method at the default settings of the Gaussian 16, which is widely employed in the description of the thermodynamic characteristics of solvation. The zero-point energies and thermal correction, together with the entropies, were used to convert the internal energies to standard Gibbs energy at 298.15 K. The NBO technique was used for electron and spin calculations in the population analyses [22].

## 4. Conclusions

In conclusion, we have investigated the reaction between electrogenerated O_2_^•−^ and 5-ASA in DMF. As a result, we have concluded that:

- 5-ASA eliminates O_2_^•−^ through PCET involving three PTs and one ET;
- the net reaction mechanism involves three steps: initial deprotonation of the 1-carboxyl group of 5-ASA, subsequent PCET between the *p*-AP moiety of the deprotonated 5-ASA anion and O_2_^•−^, and subsequent exchange of a nitrogen atom with an oxygen atom, forming a 1,4-benzoquinone radical via an intermediate 1,4-benzoquinone imine radical;
- the 1-carboxyl group plays a protective role against the oxidative decomposition of 5-ASA under the action of O_2_^•−^ in acidic solutions.

Furthermore, we have confirmed that two structural characteristics of the *p*-AP moiety are essential for the successful O_2_^•−^ elimination through the PCET mechanism involving two PTs and one ET. These are (1) an amino proton for the PT, and (2) *para*-substitution for π-conjugation.

Thermodynamic analyses could not clarify whether the PCET between the 5-ASA anion and O_2_^•−^ proceeds via a concerted or sequential pathway. However, these findings provide another mechanistic insight, in that the PT from 1-carboxyl group is not associated with the PCET even in the sequential reaction, implying that the PCET reaction between 5-ASA and O_2_^•−^ occurs via a HB complex in the concerted pathway without dissociation of the HB rather than via the sequential pathway.

Although the results presented in this manuscript are for a chemical reaction in aprotic DMF solvent rather than a biological system, the PCET theory is adaptable to biological processes involving both protic and aprotic conditions in such as a lipid bilayer. Therefore, we hope that the findings obtained in this study will provide evidence for the biological mechanistic actions of O_2_^•−^ elimination by 5-ASA.

## Figures and Tables

**Table 1:** Δ*G*° values (kJ mol^−1^, 298.15 K) for PCET between O_2_^•−^ and the four aminophenols in DMF, calculated using DFT at the B3LYP/PCM/6-311+G(d,p) level.

Compounds	PT1	PT2	PT3	PT4	ET1	ET2	ET3	Total ^1^
5-ASA anion	155.8	−198.7	428.4	−4.4	267.8	−86.7	−519.6	64.6
*p*-AP	64.5	−302.4	447.6	−10.6	332.9	−44.0	−502.3	9.7
4-ASA anion	133.8	−218.0	435.6	118.3	314.0	−37.7	−355.1	214.4
*m*-AP	47.9	−294.9	438.7	−7.0	358.1	15.2	−430.5	56.1

^1^ Total values involves sum of Δ*G*°s for two PTs and one ET.

## Data Availability

The data presented in this study are available on request from the corresponding author.

## Supplementary Materials

The following are available online at https://www.mdpi.com/1424-8247/14/2/120/s1, Figure S1: Cyclic voltammograms of O_2_/O_2_^•−^ in the presence of cyanophenol (typical phenolic compound), Figure S2: In situ electrolytic ESR system, Table S1: Charge distributions and natural population on 1,4-benzoquinone-2-carboxylic acid radical anion obtained by the NBO analysis, Table S2: Charge distributions and natural population on 5-ASA^a^, 5-ASA^b^, and deprotonated 5-ASA anion obtained by the NBO analysis.
