# Supplementary material for: Electrochemical and Mechanistic Study of Superoxide Elimination by Mesalazine through Proton-Coupled Electron Transfer
Source: Pharmaceuticals (Basel). 2021 Feb 4;14(2):120. doi: 10.3390/ph14020120 (PMC7915641; doi:10.3390/ph14020120)
Supplement: Supplementary file 1 [file pharmaceuticals-14-00120-s001.pdf]

Supplementary information for

Electrochemical and Mechanistic Study of Superoxide Elimination by Mesalazine through Proton-Coupled Electron Transfer

Tatsushi Nakayama [1,*] and Ryo Honda [2]

[1] Department of Pharmacy, Gifu Pharmaceutical University, 1-25-4, Daigaku-nishi, Gifu 501-1196, Japan
[2] United Graduate School of Drug Discovery and Medical Information Sciences, Gifu University, 1-1 Yanagido, Gifu 501-1193, Japan; ryohonda.rh@gmail.com
* Correspondence: tnakayama@gifu-pu.ac.jp; Tel.: +8158-230-8100

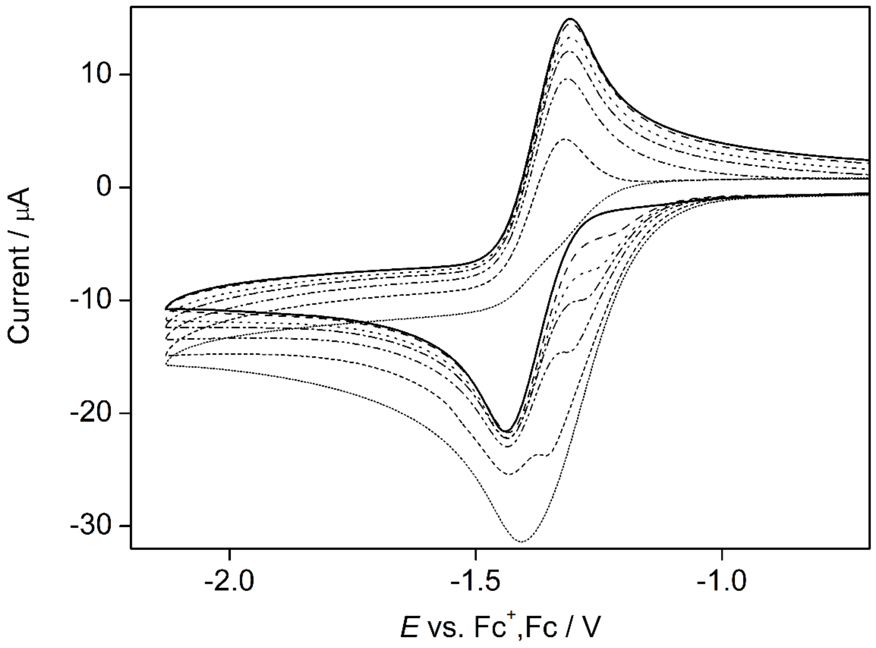

Figure S1. Cyclic voltammograms of $O_2/O_2^{\bullet-}$ in the presence of cyanophenol (acidic substrate). The voltammograms were measured in DMF containing 0.1 mol dm^{-3} tetrapropylammonium perchlorate (TPAP) on a GC electrode at a scan rate of 0.100 V s^{-1}. Concentrations (mmol dm^{-3}) of cyanophenol are 0, 1, 2, 3, 5, and 10.

CV parameter

Workin Electrode	Planar Radius 1.0 mm diameter
Geometry	Spherical (Hemispherical)[1]
Diffusion	Semi-infinite 1D
Temperature	298.3 K

[1]Operating conditions were spherical diffusion (to mimic edge diffusion to the disk)

[2]Digielch 4.5 ElchSoft inc. (Digital CV Simulation Software)

Charge Transfer Reaction			
$O_2 + e^- \leftrightarrow O_2^{\bullet -}$	Redox Potential (V vs Fc^+/Fc)	Coefficient	Kinetics (cm s^{-1})
	$E = -1.284$	$\alpha = 0.005$	$k_s = 0.00927$

Species		
	Diffusion Coefficient ($cm^2\ s^{-1}$)	Initial Concentration (mol L^{-1})
O_2	$4.76 \pm 0.24 \times 10^{-5}$	0.0048
$O_2^{\bullet -}$	$2.15 \pm 0.24 \times 10^{-5}$	0

Refferences)

Valencia D.P.; Gonzalez F.J. Estimation of diffusion coefficients by using a linear correlation between the diffusion coefficient and molecular weight. *J. Electroanalytical Chemistry* **2012**, 681, 121-126. https://doi.org/10.1016/j.jelechem.2012.06.013

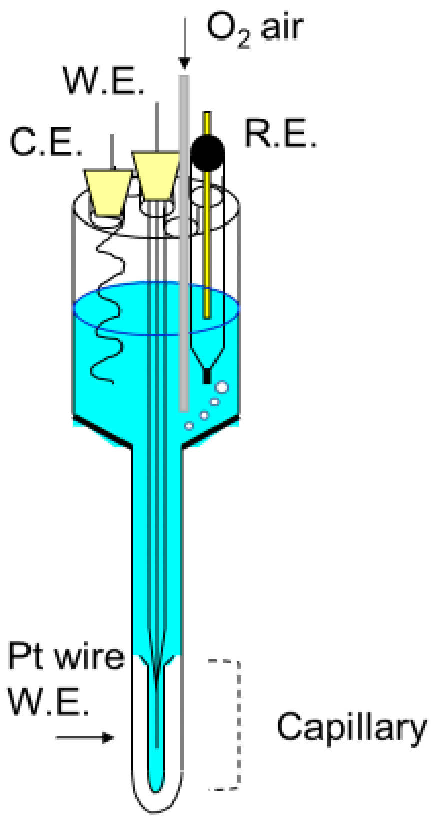

Figure S2. *In situ* electrolytic ESR system, composed of an electrochemical ESR cell with a glass small tip, air tube for O_2 bubbling, and three electrode system using a 0.5-mm-diameter straight Pt wire sealed in a glass capillary as working electrode.

Table S1. Charge distributions and natural population on 1,4-benzoquinone-2-carboxylic acid radical anion obtained by the NBO analysis (calculated with DFT-B3LYP/PCM/6-311+G(d,p) in DMF).

1,4-benzoquinone-2-carboxylic acid radical anion ================================

Atom	No	Charge	Core	Valence	Rydberg	Total
C	1	-0.22008	1.99905	4.19859	0.02244	6.22008
C	2	-0.24901	1.99905	4.22806	0.02190	6.24901
C	3	0.40593	1.99901	3.56500	0.03007	5.59407
C	4	-0.24945	1.99882	4.22799	0.02264	6.24945
C	5	-0.18091	1.99899	4.15907	0.02284	6.18091
C	6	0.35279	1.99907	3.61428	0.03386	5.64721
H	7	0.21369	0.00000	0.78446	0.00185	0.78631
H	8	0.21460	0.00000	0.78204	0.00336	0.78540
H	9	0.22209	0.00000	0.77545	0.00246	0.77791
C	10	0.81002	1.99929	3.14848	0.04220	5.18998
O	11	-0.68504	1.99974	6.65578	0.02953	8.68504
O	12	-0.73466	1.99973	6.70184	0.03308	8.73466
O	13	-0.72175	1.99976	6.69504	0.02695	8.72175
H	14	0.50118	0.00000	0.49265	0.00616	0.49882
O	15	-0.67940	1.99979	6.65318	0.02643	8.67940
Total		-1.00000	21.99230	56.68192	0.32578	79.00000

Table S2. Charge distributions and natural population on 5-ASA[a], 5-ASA[b], and deprotonated 5-ASA anion obtained by the NBO analysis (calculated with DFT-B3LYP/PCM/6-311+G(d,p) in DMF).

5-ASA[a]===

Atom	No	Charge	Core	Valence	Rydberg	Total
C	1	-0.19214	1.99909	4.17667	0.01638	6.19214
C	2	-0.24563	1.99909	4.22849	0.01805	6.24563
C	3	0.34185	1.99877	3.63360	0.02577	5.65815
C	4	-0.23331	1.99886	4.21753	0.01692	6.23331
C	5	-0.20216	1.99904	4.18672	0.01641	6.20216
C	6	0.13388	1.99900	3.84836	0.01876	5.86612

H	7	0.21972	0.00000	0.77862	0.00166	0.78028
H	8	0.22794	0.00000	0.77025	0.00182	0.77206
H	9	0.22977	0.00000	0.76819	0.00204	0.77023
C	10	0.80688	1.99929	3.14731	0.04652	5.19312
O	11	-0.67743	1.99972	6.66309	0.01462	8.67743
O	12	-0.67786	1.99974	6.66698	0.01114	8.67786
H	13	0.50709	0.00000	0.48860	0.00431	0.49291
O	14	-0.70023	1.99975	6.68654	0.01394	8.70023
H	15	0.50409	0.00000	0.49030	0.00561	0.49591
N	16	-0.81257	1.99944	5.79447	0.01866	7.81257
H	17	0.38518	0.00000	0.61266	0.00216	0.61482
H	18	0.38493	0.00000	0.61287	0.00220	0.61507
Total		0.00000	21.99178	57.77125	0.23697	80.00000

5-ASA[b]

Atom	No	Charge	Core	Valence	Rydberg	Total
C	1	-0.20989	1.99909	4.19417	0.01662	6.20989
C	2	-0.25530	1.99908	4.23893	0.01730	6.25530
C	3	0.29813	1.99870	3.68103	0.02214	5.70187
C	4	-0.20995	1.99884	4.19388	0.01724	6.20995
C	5	-0.20347	1.99904	4.18754	0.01690	6.20347
C	6	0.14547	1.99901	3.83515	0.02038	5.85453
H	7	0.22200	0.00000	0.77632	0.00168	0.77800
H	8	0.22684	0.00000	0.77145	0.00170	0.77316
H	9	0.23342	0.00000	0.76443	0.00215	0.76658
C	10	0.80505	1.99935	3.14673	0.04887	5.19495
O	11	-0.64234	1.99974	6.62880	0.01380	8.64234
O	12	-0.70066	1.99973	6.68651	0.01442	8.70066
H	13	0.51240	0.00000	0.48249	0.00511	0.48760
O	14	-0.72787	1.99972	6.71610	0.01206	8.72787
H	15	0.51174	0.00000	0.48463	0.00363	0.48826
N	16	-0.80652	1.99943	5.79146	0.01564	7.80652
H	17	0.40051	0.00000	0.59762	0.00187	0.59949
H	18	0.40046	0.00000	0.59765	0.00189	0.59954

Total	0.00000	21.99173	57.77489	0.23338	80.00000

5-ASA anion ==

Atom	No	Charge	Core	Valence	Rydberg	Total
C	1	-0.23109	1.99908	4.21503	0.01698	6.23109
C	2	-0.25834	1.99909	4.24062	0.01863	6.25834
C	3	0.30717	1.99876	3.66806	0.02601	5.69283
C	4	-0.21162	1.99884	4.19400	0.01877	6.21162
C	5	-0.22158	1.99902	4.20508	0.01748	6.22158
C	6	0.12203	1.99898	3.86019	0.01881	5.87797
H	7	0.21083	0.00000	0.78740	0.00177	0.78917
H	8	0.21623	0.00000	0.78182	0.00195	0.78377
H	9	0.22220	0.00000	0.77539	0.00241	0.77780
C	10	0.76539	1.99940	3.17496	0.06026	5.23461
O	11	-0.78035	1.99977	6.76725	0.01333	8.78035
O	12	-0.82385	1.99975	6.80946	0.01463	8.82385
O	13	-0.74575	1.99976	6.73093	0.01506	8.74575
H	14	0.49685	0.00000	0.49634	0.00681	0.50315
N	15	-0.82485	1.99945	5.80576	0.01964	7.82485
H	16	0.37873	0.00000	0.61898	0.00229	0.62127
H	17	0.37801	0.00000	0.61967	0.00232	0.62199
Total		-1.00000	21.99190	57.75096	0.25714	80.00000
